# Two new species of *Sinella* from Guangdong Province, China (Collembola: Entomobryidae)

**DOI:** 10.3897/zookeys.611.9025

**Published:** 2016-08-15

**Authors:** Guo-Liang Xu, Wei-Yu Chen

**Affiliations:** 1 School of Geographical Sciences, Guangzhou University, Guangzhou 510006, P. R. China; 2 Nanjing Plant Protection Station, No 169 Hanzhongmen Road, Nanjing 210036, P. R. China

**Keywords:** Blind species, chaetotaxy, springtail, Sinella colubra sp. n., Sinella zhangi sp. n., South China

## Abstract

Two new blind species of *Sinella* are described from Guangdong Province, China. *Sinella colubra* **sp. n.** possesses minute smooth postlabial chaetae, long mucronal spine, and 4+4(5) lateral mac on Abd. IV, and can be distinguished from two closely related species by the postlabial chaetae and the dorsal macrochaetotaxy. *Sinella zhangi* **sp. n.** is also described and can be diagnosed by having minute labial chaeta r and postlabial chaetae X and X_4_, 5+5 mac on Abd. I, 4+4 central mac on Abd. II, and 4+4 central and 5+5 lateral mac on Abd. IV.

## Introduction

The genus *Sinella* Brook, 1882 is distributed worldwide and is very abundant in China. Deharveng (1990), Chen and Christiansen (1993) and Zhang et al. (2009, 2011) made significant contributions to the modern taxonomy of the genus. Members of the genus have 4-segmented antennae, reduced eye number (0‒6 on each side), pigment reduced or absent, polymacrochaetotic chaetotaxy, bidentate mucro, and no dental spines and scales. So far, 39 species, including 14 blind ones, have been recorded from China. Among them, only two eyed species have been recorded from Guangdong Province: *Sinella curviseta* Brook, 1882 and *Sinella longisensilla* Zhang, 2013. In this study, two new blind species are described from Guangdong.

## Materials and methods

Specimens were cleared in Nesbitt’s fluid (Krantz 1978), mounted under a coverslip in Hoyer’s solution, and observed using a Nikon E80i microscope. The labial chaetae terminology follows Gisin’s system (1967). The dorsal and ventral chaetotaxy of head are described after Chen and Christiansen (1993), completed for the anterior part of head after Jordana and Baquero (2005) and Soto-Adames (2008). Dorsal body chaetae are designated following Szeptycki (1979) and Zhang et al. (2011). The number of macrochaetae is given by half-tergite in the descriptions (left side of tergites drawn in figures). Tergal S-chaetotaxic formula follows Zhang and Deharveng (2015). All descriptions are based on fully developed adults if not otherwise mentioned. Symbols representing chaetal elements used in the figures are as follows: large circle, macrochaeta; small circle, mesochaeta; cross, bothriotrichum; circle with a slash, pseudopore; dotted circle, chaetae present or absent. All materials are deposited in the collections of the Department of Entomology, College of Plant Protection, Nanjing Agricultural University (NJAU), P. R. China.

### Abbreviations:

Th.
 thoracic segment 


Abd.
 abdominal segment 


Ant.
 antennal segment 


mac
 macrochaeta/ae 


mic
 microchaeta/ae 


mes
 mesochaeta/ae 


ms
 S-microchaeta/ae 


sens
 ordinary tergal S-chaeta/ae 


## Taxonomy

### Sinella colubra sp. n.

Taxon classificationAnimaliaCollembolaEntomobryidae

http://zoobank.org/543604B9-A3BA-4FA9-ADC0-A506FCC99B04

Figs 1–12 , 13–16 , Table 1

#### Type material.

Holotype: ♂ on slide, China, Guangdong Province, Huizhou City, Longmen County, Nankunshan Natural Reserve, 23°38'4.01”N, 113°51'15.25”E, altitude 497 m, 24 August 2010, Z-X Pan and Y-T Ma leg. (# S4143). Paratypes: ♂ and 3 ♀♀ on slides and 3 in alcohol, same data as holotype.

#### Other material.

♀ on slide, China, Guangdong Province, Nanling National Natural Reserve, 24°55'42.6"N, 113°0'58.3"E, altitude 1026 m, 22 July 2010, F Zhang and Z-H Li leg. (# C9640).

#### Diagnosis.

No eyes. Long smooth straight chaetae present on antennae. Clypeal chaetae eight and median three much smaller. Labial chaetae as mrel_1_l_2_. Postlabial chaetae X and X_2‒4_ minute. “Smooth” inner differentiated tibiotarsal chaetae present. Manubrium without smooth chaetae. Mucronal spine long, with tip nearly reaching apical tooth. Abd. I with 6+6 mac. Abd. II with 3+3 central mac. Abd. IV with 7+7 central and 4+4 lateral mac.

**Table 1.** Comparison among *Sinella colubra* **sp. n.**, *Sinella insolens* and *Sinella sineocula*. Rare character states are noted and placed in parentheses.

**Characters**	***Sinella colubra* sp. n.**	***Sinella insolens***	***Sinella sineocula***
Mac in Gr. II on dorsal head	3(4)	usually 4‒5(6‒8)	usually 5‒6(4)
Labial chaeta M1s	absent	present	absent
Postlabial chaetae X and X_2‒4_	smooth, minute	cliate, large	ciliate, large
Mac m5i on Th. III	absent	present	absent
Mac on Abd. I	6+6	7(6)+7(6)	7(6)+7(6)
Central mac on Abd. II	3+3	4+4	3+3
Lateral mac on Abd. IV	4(5)+4(5)	6+6	6+6

#### Description.

Body length up to 1.50 mm. Body pale in alcohol.

Antenna 1.63‒1.93 times as long as cephalic diagonal. Antennal segments ratio as I : II : III : IV = 1 : 1.77‒2.00 : 1.64‒1.74 : 2.57‒2.91. Smooth spiny mic at base of antennae: three dorsal, three ventral on Ant. I; one internal, one external and two ventral on Ant. II. Ant. II distally with one (rarely two) rod-like sens. Ant. III organ with two slightly expanded internal sens. Ant. IV with a knobbed subapical organ. Ant. II. with 2‒4 ventral long smooth straight chaetae.

**Figures 1–12.**
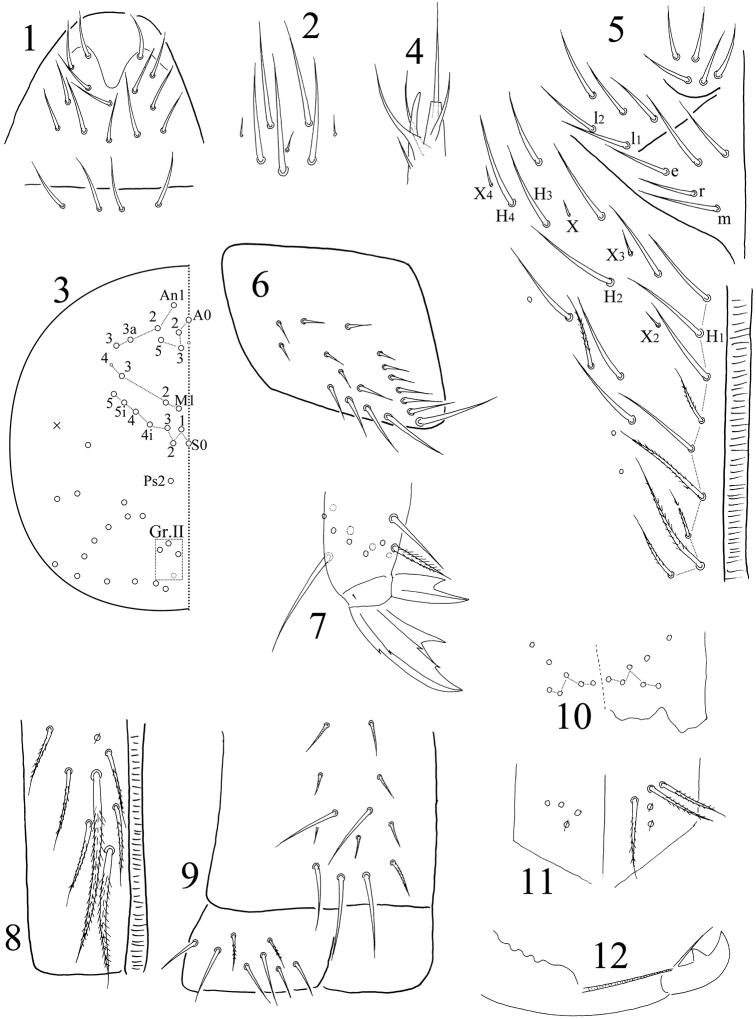
*Sinella colubra* sp. n. **1** labrum **2** clypeal chaetae **3** dorsal cephalic chaetotaxy **4** lateral process and labial papilla E **5** chaetae on the ventral side of head **6** trochanteral organ **7** hind claw **8** anterior face of ventral tube **9** ventral face and lateral flap of ventral tube **10** distal part of anterior face of manubrium **11** manubrial plaque **12** mucro.

Eyes absent in all specimens. Prelabral and labral chaetae 4/ 5, 5, 4, all smooth; labral intrusion U-shaped (Fig. 1). Clypeal chaetae eight in number, including three small median chaetae (Fig. 2). Dorsal cephalic chaetotaxy with four antennal (An), three median (M) and eight sutural (S) mac; Gr. II with 3(4) mac (Fig. 3). Mandibles with 4/5 (left/right) teeth. Subapical chaeta of maxillary outer lobe slightly thicker than apical one; three smooth sublobal hairs on maxillary outer lobe. Lateral process of labial palp slightly thicker than normal chaetae, with tip extending beyond apex of labial papilla (Fig. 4). Labial chaetae as mrel_1_l_2_, all smooth, r/m=0.67‒0.79; chaetae X and X_2‒4_ minute; chaeta X_3_ rarely absent; H_1-4_ smooth. Cephalic groove with 9(8) chaetae, four of them smooth and others ciliate (Fig. 5).

Trochanteral organ with 17‒19 smooth spiny chaetae; 11–12 in arms and 5‒7 internal (Fig. 6). Partial inner differentiated tibiotarsal chaetae “smooth” with ciliations closely appressed to axis (Chen and Christiansen 1993). Tibiotarsi distally with ten chaetae in a whorl. Unguis with three inner, one outer, and two lateral teeth; two paired teeth unequal, outer one large. Unguiculus with a large outer tooth. Tenent hairs of all legs pointed but clavate in one male specimen (Fig. 7). Abd. IV 3.72‒4.60 times as long as Abd. III along dorsal midline. Ventral tube anteriorly with 6‒7 ciliate chaetae on each side, two of them much larger than others (Fig. 8); posteriorly with about 13 chaetae, most of them small and weakly ciliate; each lateral flap with two ciliate and six smooth chaetae (Fig. 9). Male genital plate not clearly seen. Manubrium dorsally without smooth chaetae; ventrally with 5+5 distal ciliate chaetae (Fig. 10). Manubrial plaque with 2+2(1) pseudopores and 3+3 ciliate chaetae (Fig. 11). Distal smooth part of dens 1.08–1.57 as long as mucro. Mucro bidentate with apical tooth larger; basal spine long, with tip nearly reaching apical tooth (Fig. 12).

**Figures 13–16.**
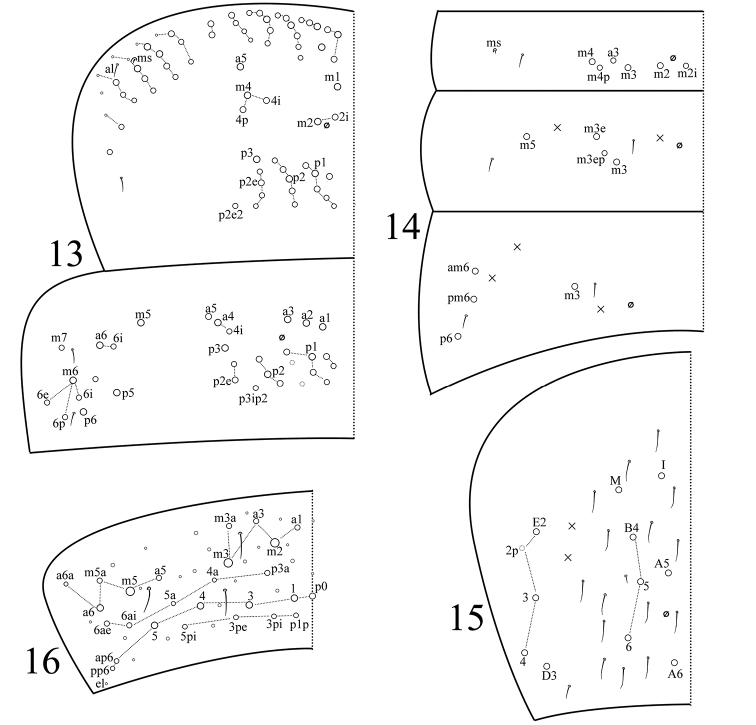
Tergal chaetotaxy of *Sinella colubra* sp. n. **13** thorax **14** Abd. I‒III **15** Abd. IV **16** Abd. V.

Th. II with three medio-medial mac (m1, m2, m2i), three medio-lateral mac (m4, m4i, m4p), 14‒18 posterior mac, one ms and two sens; ms interior to sens al. Th. III with 29‒32 mac and two lateral sens; a6i, p5, p6, m6, m6i, m6p, m6e and m6ai2 present as mac; mac m5i absent (Fig. 13). Abd. I with six mac (a3, m2–4, m2i, m4p), one ms and one sens; sens interior to ms. Abd. II with three central mac (m3, m3e, m3ep), one lateral mac (m5) and two sens. Abd. III with one central mac (m3), three lateral mac (am6, pm6, p6) and two sens; ms absent (Fig. 14). Abd. IV with seven central mac (I, M, A5–6, B4–6), four (rarely five) lateral mac (D3, E2–4), and at least 17 sens (Fig. 15). Abd.V with three sens (Fig. 16).

#### Etymology.

Named after the snake *Bungarus multicinctus* Blyth found in the sampling site.

#### Ecology.

In soil of bamboo forest, near termitarium.

#### Remarks.

*Sinella colubra* sp. n. is most similar to *Sinella insolens* Chen & Christiansen, 1993 and *Sinella sineocula* Chen & Christiansen, 1993 in morphology of unguis and unguiculus, long mucronal spine, absence of smooth chaetae on manubrium, medial and posterior mac on Th. II, 1+1 central and 3+3 lateral mac on Abd. III, and 7+7 central mac on Abd. IV. It differs from them in 3+3(4) cephalic mac on Gr. II, absence of labial chaeta M1s, minute postlabial chaetae X and X_2‒4_, absence of mac m5i on Th. III, 6+6 (a2 as mes) mac on Abd. I, absence of mac m3ei on Abd. II, and 4+4(5) lateral mac on Abd. IV.

### Sinella zhangi sp. n.

Taxon classificationAnimaliaCollembolaEntomobryidae

http://zoobank.org/E8161EA4-8F9E-4C9C-8A90-C9CFEB9E8A01

Figs 17–25 , 26–27

#### Type material.

Holotype: ♂ on slide, China, Guangdong Province, He Mountain, in soil of secondary eucalypt forest, 22 October 2012, Guoliang Xu leg. (# Xu-2012). Paratypes: 1 ♀ on slide and 1 juvenile in alcohol, same data as holotype.

#### Diagnosis.

No eyes. Long smooth straight chaetae absent on antennae. Labial chaeta r and postlabial chaetae X and X_4_ minute. “Smooth” inner differentiated tibiotarsal chaetae present. Tenent hairs clavate. Manubrium without smooth chaetae. Mucronal spine short, with tip reaching subapical tooth. Chaeta p5 as mac on Th. II. Abd. I with 5+5 mac. Abd. II with 4+4 central mac. Abd. IV with 4+4 central and 5+5 lateral mac.

#### Description.

Body length up to 1.32 mm. Body pale in alcohol.

Antenna 2.04 times as long as cephalic diagonal. Antennal segments ratio as I : II : III : IV = 1 : 1.71 : 1.86: 2.71. Smooth spiny mic at base of antennae three dorsal, three ventral on Ant. I: one internal, one external and one ventral on Ant. II. Ant. II distally with one rod-like S-chaeta. Two internal sens of Ant. III organ rod-like. Long smooth straight chaetae absent on antennae.

**Figures 17–25.**
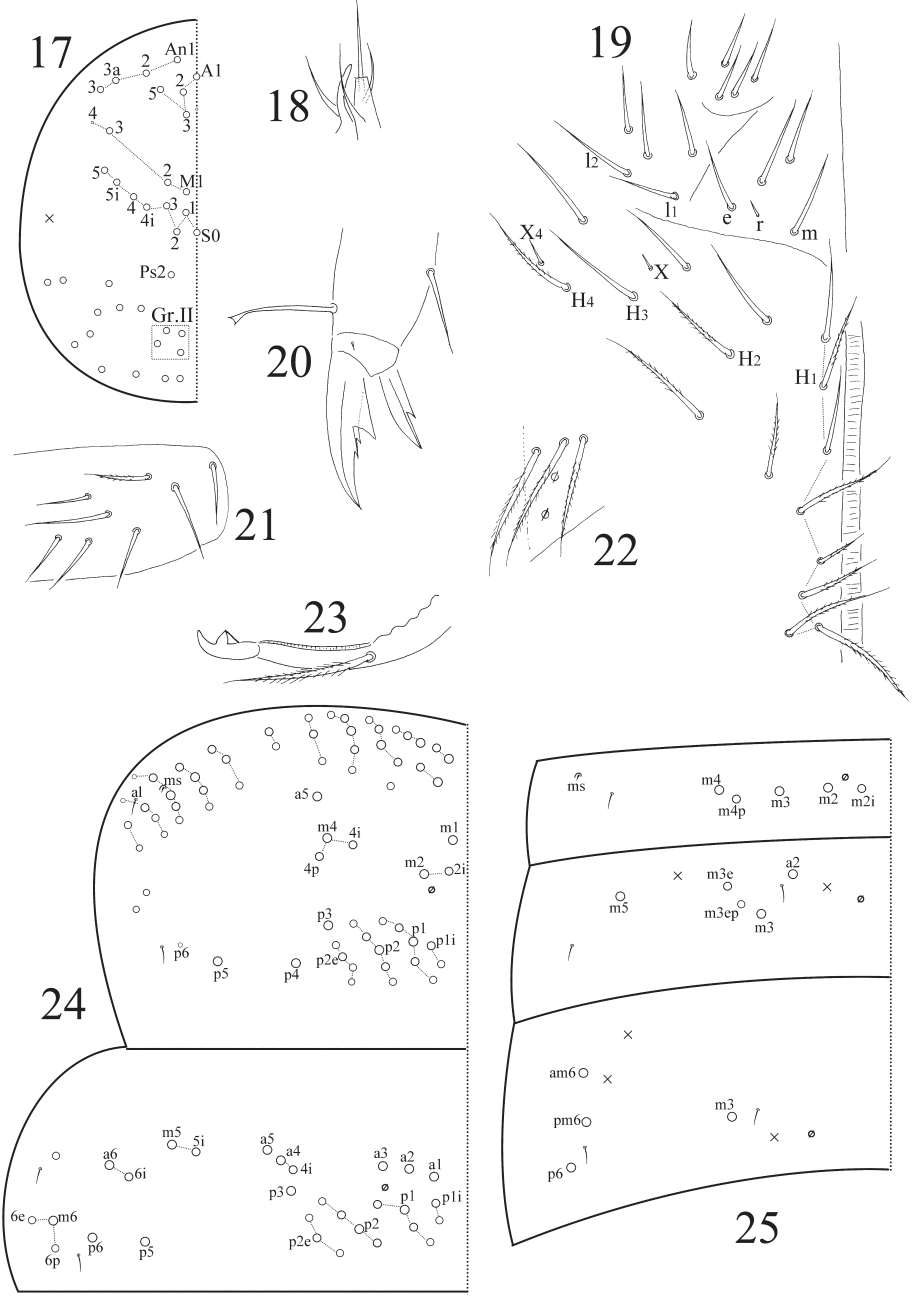
*Sinella zhangi* sp. n. **17** dorsal cephalic chaetotaxy **18** lateral process and labial papilla E **19** chaetae on the ventral side of head **20** hind claw **21** lateral flap of ventral tube **22** manubrial plaque **23** mucro **24** thoracic chaetotaxy **25** chaetotaxy of Abd. I‒III.

Eyes absent in all specimens. Prelabral and labral chaetae 4/ 5, 5, 4, all smooth; three chaetae of first row longer than lateral chaetae. Clypeal chaetae not clearly seen. Dorsal cephalic chaetotaxy with four antennal, three median (M) and eight sutural (S) mac; Gr. II with four mac (Fig. 17). Mandibles with 4/5 (left/right) teeth. Subapical chaeta of maxillary outer lobe subequal to apical chaeta; three smooth sublobal hairs on maxillary outer lobe. Lateral process of labial palp thicker than normal chaetae, with tip extending beyond apex of labial papilla (Fig. 18). Labial chaetae as mrel_1_l_2_, all smooth, r/m=0.20; chaetae X and X_4_ smooth, minute; chaetae X_2‒3_ absent; H_1_, H_2_ and H_4_ ciliate. Cephalic groove with eight chaetae, two smooth and others ciliate (Fig. 19).

Trochanteral organ with nine smooth spiny chaetae; five in arms and four internal. Some inner differentiated tibiotarsal chaetae “smooth” with ciliations closely appressed to axis. Tibiotarsi distally with ten chaetae in a whorl. Unguis with three inner teeth; two paired teeth unequal, outer one larger. Unguiculus with a large outer tooth. Tenent hairs clavate (Fig. 20). Abd. IV 3.42 times as long as Abd. III along dorsal midline. Ventral tube anteriorly with seven ciliate chaetae; two of them much larger than others; posteriorly not clearly seen; each lateral flap with seven smooth and one ciliate chaetae (Fig. 21). Manubrium without smooth chaetae. Manubrial plaque with 2+2 pseudopores and 3+3 ciliate chaetae (Fig. 22). Distal smooth part of dens 1.72 times as long as mucro. Mucro bidentate with apical tooth longer than subapical tooth; basal spine short, reaching tip of subapical tooth (Fig. 23).

**Figures 26–27.**
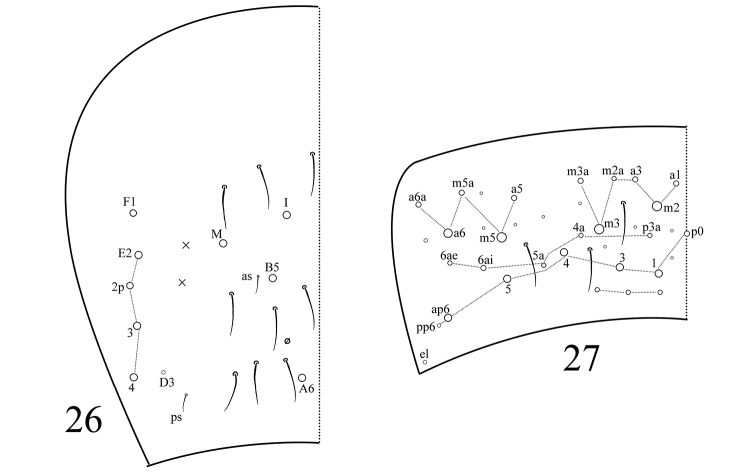
Tergal chaetotaxy of *Sinella zhangi* sp. n. **26** Abd. IV **27** Abd. V.

Th. II with three medio-medial mac (m1, m2, m2i), three medio-lateral mac (m4, m4i, m4p), 19 posterior mac, one ms and two sens; ms interior to sens al. Th. III with 30 mac and two lateral sens (Fig. 24). Abd. I with five mac (m2–4, m2i, m4p), one ms and one sens; sens interior to ms. Abd. II with four central mac (a2, m3, m3e, m3ep), one lateral mac (m5) and two sens. Abd. III with one central mac (m3), three lateral mac (am6, pm6, p6) and two sens; ms absent (Fig. 25). Abd. IV with four central mac (I, M, B5, A6), five lateral mac (E2–4, E2p, F1), and at least 11 sens; as and ps shorter than others (Fig. 26). Abd.V with three sens; chaetae m2, m3, m5, a6, p1, p3–5 and ap6 present as mac (Fig. 27).

#### Etymology.

Named after the Chinese collembologist Dr. Feng ZHANG, who has made great contributions to the taxonomy of *Sinella*.

#### Ecology.

In decomposing leaves along the roads.

#### Remarks.

*Sinella zhangi* sp. n. is most similar to *Sinella quadriseta* Zhang, Bedos & Deharveng, 2014 in absence of eyes, tip of lateral process of labial palp beyond apex of labial papilla, morphology of unguis and unguiculus, and mucronal spine, but differs from it in smooth, minute labial and postlabial chaetae r, X and X_4_, clavate tenent hairs, “smooth” inner differentiated tibiotarsal chaetae, 4+4 mac in Gr. II on dorsal head, absence of mac m2, m2i, m4i and m4p on Th. II, 5+5 mac on Abd. I (m4p present), and 4+4 central mac on Abd. II (m3ep present). It is also similar to Chinese species *Sinella affluens* Chen & Christiansen, 1993 in 4+4 mac on dorsal head, “smooth” inner differentiated tibiotarsal chaetae, clavate tenent hairs, number of teeth on unguis and unguiculus, minute postlabial chaetae X and X_4_, 2+2 pseudopores and 3+3 ciliate chaetae on manubrial plaque, medial mac on Th. II, 1+1 central mac on Abd. III, and 4+4 central mac on Abd. IV, but differs from the latter in absence of eyes, short mucronal spine, minute labial chaeta r, p5 present as mac on Th. II, m5i present as mac on Th. III, 5+5 mac on Abd. I (a3) as mes, 4+4 central mac on Abd. II (a2 as mac), 5+5 lateral mac on Abd. IV (F1 as mac).

## Supplementary Material

XML Treatment for Sinella colubra

XML Treatment for Sinella zhangi

## References

[B1] Brook G (1882) On a new genus of Collembola (*Sinella*) allied to *Degeeria* Nicolet. Journal of the Linnean Society of London Zoology 16: 541–545. doi: 10.1111/j.1096-3642.1882.tb02398.x

[B2] Chen J-X Christiansen KA (1993) The genus *Sinella* with special reference to *Sinella* *s. s.* (Collembola: Entomobryidae) of China. Oriental Insects 27: 1–54. doi: 10.1080/00305316.1993.10432236

[B3] Deharveng L (1990) Fauna of Thai caves. II. New Entomobryoidea Collembola from Chiang Dao cave, Thailand. Occasional Papers of the Bernice P. Bishop Museum 30: 279–287. http://hbs.bishopmuseum.org/pubs-online/pdf/op30p279.pdf

[B4] Gisin H (1967) Espèces nouvelles et lignées évolutives de *Pseudosinella* endogés (Collembola). Memórias e Estudos do Museu Zoológico da Universidade de Coimbra 301: 1–25.

[B5] Jordana R Baquero E (2005) A proposal of characters for taxonomic identification of *Entomobrya* species (Collembola, Entomobryomorpha), with description of a new species. Abhandlungen und Berichte des Naturkundemuseums Görlitz 76: 117–134.

[B6] Krantz GW (1978) A manual of Acarology. 2nd edition. Oregon State University Book Store, Corvallis, Oregon, 509 pp.

[B7] Soto-Adames FN (2008) Postembryonic development of the dorsal chaetotaxy in *Seira dowlingi* (Collembola, Entomobryidae); with an analysis of the diagnostic and phylogenetic significance of primary chaetotaxy in *Seira*. Zootaxa 1683: 1–31.

[B8] Szeptycki A (1979) Morpho-systematic studies on Collembola. IV. Chaetotaxy of the Entomobryidae and its phylogenetical significance. Polska Akademia Nauk, Kraków, 219 pp.

[B9] Zhang F (2013) Five new eyed species of *Sinella* (Collembola: Entomobryidae) from China, with a key to the eyed species of the genus. Zootaxa 3736: 549–568. doi: 10.11646/zootaxa.3736.5.7 25112646 10.11646/zootaxa.3736.5.7

[B10] Zhang F Bedos A Deharveng L (2014) New species of *Sinella* and *Coecobrya* (Collembola: Entomobryidae) from New Caledonia. Zootaxa 3814: 553–566. doi: 10.11646/zootaxa.3814.4.7 24943448 10.11646/zootaxa.3814.4.7

[B11] Zhang F Deharveng L (2015) Systematic revision of Entomobryidae (Collembola) by integrating molecular and new morphological evidence. Zoologica Scripta 44: 298–311. doi: 10.1111/zsc.12100

[B12] Zhang F Deharveng L Chen J-X (2009) New species and rediagnosis of *Coecobrya* (Collembola: Entomobryidae), with a key to the species of the genus. Journal of Natural History 43: 2597–2615. doi: 10.1080/00222930903243970

[B13] Zhang F Yu D-Y Xu G-L (2011) Transformational homology of the tergal setae during postembryonic development in the *Sinella*-*Coecobrya* group (Collembola: Entomobryidae). Contributions to Zoology 80: 213–230. http://www.ctoz.nl/cgi/t/text/get-pdf?c=ctz;idno=8004a01

